# In Vitro Antidiabetic Activity of Phenolic Compounds from NADES-Extracted SunGold Kiwifruit and Their Fortification in Yoghurt

**DOI:** 10.3390/foods15173109

**Published:** 2026-09-01

**Authors:** Rifqah Azzahra Naulidia, Michelle Ji Yeon Yoo

**Affiliations:** School of Science, Faculty of Health and Environmental Sciences, Auckland University of Technology, Private Bag 92006, Auckland 1142, New Zealand; mmt1991@autuni.ac.nz

**Keywords:** natural deep eutectic solvent, SunGold kiwifruit, functional yoghurt, phenolic compounds, α-glucosidase inhibition, α-amylase inhibition, glucose diffusion, antidiabetic activity

## Abstract

Type 2 diabetes mellitus (T2DM) is a major global health challenge, which has increased interest in functional foods containing bioactive compounds with antidiabetic potential. This study evaluated the in vitro antidiabetic activity of phenolic compounds extracted from SunGold kiwifruit using a choline chloride–glycerol natural deep eutectic solvent (NADES) and their application in yoghurt. NADES and water extracts were assessed for α-glucosidase and α-amylase inhibition and glucose diffusion retardation. The extracts were incorporated into yoghurt at different concentrations before or after fermentation, followed by assessment of enzyme-inhibitory activities and phenolic profiles. The NADES extract showed stronger α-glucosidase and α-amylase inhibition than the water extract, with IC_50_ values of 15.331 ± 0.393 and 116.530 ± 2.110 mg/mL, respectively. It also produced greater glucose diffusion retardation at 180 min (62.84%) than the water extract (37.89%). Fortification enhanced enzyme-inhibitory activities in a concentration-dependent manner, with post-fermentation yoghurt containing 30% NADES extract showing the highest α-glucosidase and α-amylase inhibitory activities of 3.615 ± 0.032 mg QE/g and 111.186 ± 0.541 mg AE/g, respectively. LC-MS/MS identified catechin, epicatechin, epigallocatechin, chlorogenic acid, caffeic acid, and gallic acid. These findings demonstrate that the NADES preparation showed greater in vitro carbohydrate-digestive enzyme inhibition and glucose diffusion retardation than the water extract under the tested conditions.

## 1. Introduction

Type 2 diabetes mellitus (T2DM) has emerged as one of the most pressing public health challenges worldwide. Its prevalence continues to increase in both developed and developing countries, driven by ageing populations, urbanisation, and lifestyle-related factors such as high-calorie diets, reduced physical activity, and increasing obesity rates [1]. According to the International Diabetes Federation, approximately 589 million adults aged 20–79 years were living with diabetes in 2024, and this number is projected to rise to 853 million by 2050 [2]. Although conventional pharmacological therapies, including metformin, sulfonylureas, and insulin, are effective in controlling blood glucose, their long-term use may be associated with side effects, reduced therapeutic efficacy, and limited patient adherence [3]. Therefore, alternative or complementary dietary strategies for the prevention and management of T2DM are urgently needed.

Functional foods have gained increasing attention as a practical dietary approach to support diabetes management. Unlike pharmaceutical treatments, functional foods can be incorporated into daily eating habits and may provide bioactive compounds with potential health benefits in a convenient and acceptable form. Among various food matrices, yoghurt is particularly attractive due to its high protein content, nutritional value, wide consumer acceptance, and compatibility with bioactive ingredient fortification [4]. A previous study has also reported that yoghurt consumption is associated with a reduced risk of T2DM, particularly among older adults, a population group more vulnerable to metabolic disorders [5]. Therefore, incorporating plant-derived phenolic extracts into yoghurt may provide a simple and acceptable strategy to develop functional foods with potential antidiabetic properties.

SunGold kiwifruit (*Actinidia chinensis* var. *chinensis*) is a relatively recent kiwifruit cultivar and is recognised as a nutrient-rich fruit containing vitamin C, dietary fibre, organic acids, and various phenolic compounds with potential metabolic health benefits [6]. Previous studies have reported that kiwifruit phenolic compounds, including catechin, epicatechin, chlorogenic acid, and procyanidin B2, are associated with antioxidant activity and inhibition of carbohydrate-hydrolysing enzymes [7]. Phenolic-rich extracts from thinned young kiwifruit have also shown strong α-glucosidase inhibitory activity, with IC_50_ values ranging from 23.82 to 62.98 µg/mL, indicating their potential relevance for glycaemic management [8]. However, studies on the incorporation of kiwifruit phenolic extracts into food matrices, particularly yoghurt, remain limited.

Prior to food incorporation, the extraction method plays an important role in determining the recovery, composition, and bioactivity of phenolic compounds. Since the antidiabetic activity of phenolics may depend on specific structural characteristics and compound polarity [9], the use of a tunable extraction solvent may improve the selective recovery of phenolic compounds associated with carbohydrate-hydrolysing enzyme inhibition. Natural deep eutectic solvents (NADES) have gained increasing attention as green extraction solvents due to their adjustable polarity, low volatility, and high solubilisation capacity for plant bioactive compounds. NADES can be designed by combining different hydrogen bond acceptors and donors, allowing their physicochemical properties to be tailored toward target phenolic compounds [10].

In this study, a NADES composed of choline chloride and glycerol was used to extract phenolic compounds from SunGold kiwifruit. The resulting extract was incorporated into yoghurt to evaluate its potential as a functional food ingredient with in vitro antidiabetic activity. Specifically, this study compared NADES and water extracts for α-glucosidase inhibition, α-amylase inhibition, and glucose diffusion retardation activity, and further assessed the effects of extract type, fortification concentration, and fortification stage on the antidiabetic activity and phenolic profile of fortified yoghurt.

## 2. Materials and Methods

### 2.1. Chemicals and Reagents

Choline chloride, glycerol, α-glucosidase, α-amylase, p-nitrophenyl-α-D-glucopyranoside, quercetin, acarbose, soluble starch, iodine, glucose, methanol, hydrochloric acid, and other analytical-grade reagents were purchased from Sigma-Aldrich (St. Louis, MO, USA). Gallic acid, chlorogenic acid, caffeic acid, catechin, epicatechin, epigallocatechin, and quercetin were used as LC-MS/MS reference standards. HPLC-grade acetonitrile, formic acid, and water were used for chromatographic analysis.

### 2.2. Ultrasound-Assisted NADES Extraction of SunGold Kiwifruit Polyphenols

A NADES composed of choline chloride and glycerol at a 1:1 molar ratio, with 50% (*w*/*w*) water addition, was mixed with SunGold kiwifruit (Seeka Limited, Auckland, New Zealand) powder at a solid-to-liquid ratio of 1:20 (*w*/*v*). Ultrasonic-assisted extraction was performed in an ultrasonic bath for 20 min at 30–40 °C. For comparison, aqueous extraction was performed under the same conditions using distilled water as the extraction solvent. The resulting liquid extracts were collected and used for yoghurt fortification.

A conventional gravimetric extraction yield was not determined because the non-volatile choline chloride and glycerol remained in the NADES extract after water removal. Consequently, the resulting residue contained both the NADES matrix and kiwifruit-derived constituents, and its total mass would overestimate the amount of extracted plant material. Instead, selected individual phenolic compounds were quantified using LC-MS/MS to compare the phenolic profiles and extraction selectivity of NADES and water.

### 2.3. Fortification of Yoghurt with NADES-Extracted SunGold Kiwifruit Polyphenols

Fortification with NADES and water extracts was performed at concentrations of 10%, 20%, and 30% (*v*/*v*). The yoghurt base was prepared by reconstituting 200 g of skim milk powder (Woolworths, Auckland, New Zealand) in 800 g of boiling water (100 °C). After cooling to 46 °C, the mixture was inoculated with 2% (*w*/*w*) commercial yoghurt containing starter cultures (De Winkel, Fonterra Brands (NZ) Ltd., Auckland, New Zealand).

For pre-fermentation (PRE) treatments, the extracts were added after inoculation but before incubation. The mixtures were incubated at 43 °C for 9 h until the pH decreased below 5. For post-fermentation (POS) treatments, the extracts were added after incubation during homogenisation. An unfortified yoghurt prepared under the same conditions served as the control. All samples were homogenised at 4000 rpm for 2 min using a laboratory mixer, stored at 4 °C, and analysed within 48 h of production to minimise sample degradation [11,12].

### 2.4. Antidiabetic Activity

Antidiabetic activity was evaluated through α-glucosidase inhibition, α-amylase inhibition, and in vitro glucose diffusion assays. All three assays were performed on the raw NADES and water extracts, whereas the fortified yoghurt samples were evaluated using only the α-glucosidase and α-amylase inhibition assays.

Before analysis, the liquid extracts and fortified yoghurt samples were freeze-dried for 48 h. Freeze-drying removed the water fraction but did not remove the non-volatile choline chloride and glycerol components of the NADES. The resulting NADES residue therefore contained the NADES matrix together with the extracted kiwifruit constituents. The concentrations reported in mg/mL were calculated from the total mass of the freeze-dried NADES-containing preparation divided by the final reconstitution volume and did not represent the isolated dry mass of kiwifruit-derived compounds.

Polyphenols were recovered from the freeze-dried yoghurt samples using acidified methanol containing concentrated HCl at a sample-to-solvent ratio of 1:3 (*w*/*v*). The mixtures were homogenised at 12,000 rpm for 1 min and stored at −20 °C overnight. Subsequently, the resulting slurries were centrifuged at 10,000 rpm for 10 min at 4 °C and filtered through 0.22 µm syringe filters. The filtrates were collected and used for the enzyme inhibition assays [13,14].

#### 2.4.1. α-Glucosidase Inhibition Assay

The in vitro α-glucosidase inhibitory assay was performed using a previously reported method with slight modifications [15]. Quercetin was used as the standard reference due to its known antidiabetic activity [16]. A stock solution of quercetin (4000 µg/mL) was prepared and diluted with dimethyl sulfoxide (DMSO) to obtain the required concentrations. The raw extracts were similarly prepared at different concentrations for IC_50_ determination, whereas the yoghurt filtrates were analysed directly without further dilution. A corresponding plain NADES control, consisting of choline chloride and glycerol at a 1:1 molar ratio with 50% water but without kiwifruit, was analysed under matched assay conditions. The absorbance of the plain NADES control was subtracted from that of the corresponding NADES extract to correct for the contribution of the NADES matrix.

Briefly, 5 µL of standards, raw extracts, or yoghurt filtrate were transferred into a test tube. Then, 250 µL of 5 mM *p*-nitrophenyl-α-glucopyranoside (*p*-NPG) and 495 µL of 0.1 M phosphate buffer (pH 7.0) were added. The mixture was then incubated at 37 °C in a water bath for 5 min. Subsequently, 250 µL of α-glucosidase enzyme solution (0.062 U/mL) was added to initiate the reaction. For the blank, the enzyme solution was replaced with 250 µL of phosphate buffer. This reaction was incubated for 15 min before being terminated by adding 1 mL of 0.2 M Na_2_CO_3_. The release of *p*-nitrophenol was measured by recording the absorbance at 400 nm using a UV-Vis spectrophotometer (GENESYS™ 150 Vis/UV-Vis Spectrophotometer, Thermo Fisher Scientific, Waltham, MA, USA) [15]. The percentage inhibition was calculated using the following equation:

(1) $$\%\mathrm{Inhibition}=\frac{\left(Ablank-Asample\right)}{\left(Ablank\right)}\times 100$$

where *Ablank* represents the absorbance of the blank and *Asample* represents the absorbance of the sample. For raw extracts, the results were expressed as both IC_50_ and milligrams of quercetin equivalent per gram of sample (mg QE/g). The IC_50_ values were determined separately for each extract and reference inhibitor from their respective concentration–response curves. In contrast, quercetin-equivalent inhibitory activities were calculated by converting the corrected sample response using the quercetin calibration equation, followed by correction for the dilution factor, assay volume, and sample mass. Therefore, the values expressed as mg QE/g were not derived from ratios between the IC_50_ values.

#### 2.4.2. α-Amylase Inhibition Assay

Sample preparation was performed as described for the α-glucosidase inhibition assay (Section 2.4.1). In this procedure, 1 mL of acarbose standard, raw extract, or yoghurt filtrate was pipetted into a clean tube test. Then, 20 µL of α-amylase solution (10 mg/mL) and 400 µL of phosphate buffer (pH 6.9) were added, followed by incubation at 37 °C for 10 min in a water bath. Subsequently, 200 µL of 1% starch solution (*w*/*v*) was added, and the mixture was further incubated at 37 °C for 1 h. The reaction was then terminated by adding 200 µL of 1% iodine solution and 10 mL of distilled water. The absorbance was measured at 565 nm using a UV-Vis spectrophotometer (GENESYS™ 150 Vis/UV-Vis Spectrophotometer, Thermo Fisher Scientific, Waltham, MA, USA) [17].

For the blank, the sample and starch solution were replaced with equivalent volumes of phosphate buffer. Corresponding sample and reagent blanks were prepared by replacing the enzyme solution with phosphate buffer. Percentage inhibition was calculated using Equation (1) described in Section 2.4.1. The α-amylase inhibitory activity of the raw extracts was expressed as both IC_50_ and milligrams of acarbose equivalent per gram of sample (mg AE/g), whereas that of the fortified yoghurt samples was expressed as mg AE/g. Acarbose-equivalent inhibitory activities were calculated by converting the corrected sample response using the acarbose calibration equation, followed by correction for the dilution factor, assay volume, and sample mass. These values were calculated independently of the IC_50_ values.

#### 2.4.3. In Vitro Glucose Diffusion Retardation Assay

NADES and water extracts (250 mg) were separately mixed with 25 mL of 20 mmol/L glucose solution and transferred into cellulose dialysis tubing with a molecular-weight cut-off of 12,000 Da and a flat width of 35 mm (1.4 in) (Sigma-Aldrich, St. Louis, MO, USA). Before use, the dialysis tubing was soaked in distilled water. The sealed dialysis bags were immersed in 200 mL of distilled water and incubated at 37 °C in a shaker incubator at 120 rpm. A control containing 25 mL of 20 mmol/L glucose solution without extract was prepared under identical conditions. Aliquots (1.5 mL) were collected from the external medium at 30, 60, 120, and 180 min without volume replacement [18]. All experiments were independently performed in triplicate. The glucose dialysis retardation index (GDRI) was calculated according to the following formula:

(2) $$\mathrm{GDRI}\;(\%)=1-\frac{\left(Csample\right)}{Ccontrol}\times 100$$

where $C_{sample}$ and $C_{control}$ represent the glucose concentrations in the external media of the extract-containing and glucose-only systems, respectively, at the corresponding sampling time. Glucose concentrations were determined by HPLC using a Luna NH2 column (4.6 × 250 mm) (Shimadzu, Kyoto, Japan).

### 2.5. Quantification of Phenolic Compounds Using LC-MS/MS

Phenolic compounds in the NADES and water extracts and fortified yoghurt samples were analysed using a Stellar mass spectrometer coupled to a Vanquish UHPLC system (Thermo Fisher Scientific, San Jose, CA, USA). Before analysis, the NADES extract was diluted tenfold and subjected to solid-phase extraction (SPE) to separate the polyphenol fraction from the NADES components. The yoghurt samples were extracted with acidified methanol containing 0.1% (*v*/*v*) formic acid at a sample-to-solvent ratio of 1:3 (*w*/*v*).

Chromatographic separation was performed on an Agilent EC-C18 column (Agilent Technologies, Santa Clara, CA, USA) (2.1 × 150 mm, 2.7 µm, 100 Å). The mobile phases consisted of water containing 0.1% (*v*/*v*) formic acid (solvent A) and acetonitrile containing 0.1% (*v*/*v*) formic acid (solvent B). Gradient elution was performed over a total run time of 40 min at a flow rate of 0.30 mL/min, with an injection volume of 1 µL. Mass spectrometric detection was performed using heated electrospray ionisation (H-ESI) and targeted MS/MS analysis in negative ion mode. Gallic acid, epigallocatechin, catechin, chlorogenic acid, caffeic acid, quercetin, and epicatechin were identified using their respective reference standards. Quantification was performed using external calibration based on chromatographic peak area, and the results were expressed as μg/g of sample.

### 2.6. Statistical Analysis

All measurements were performed in at least triplicate (n ≥ 3), and the results were expressed as mean ± standard deviation (SD). Statistical comparisons among samples were performed using one-way analysis of variance (ANOVA) in Minitab Statistical Software version 22 (Minitab LLC, State College, PA, USA), followed by Tukey’s post hoc test for multiple comparisons. Three-way ANOVA was carried out on the fortified yoghurt samples, with extract type, fortification stage, and extract concentration as fixed factors, including their interaction effects. Statistical significance was set at *p* < 0.05.

## 3. Results and Discussion

### 3.1. Antidiabetic Enzyme-Inhibitory Activities of SunGold Kiwifruit Extracts

The inhibitory activities of the SunGold kiwifruit extracts against α-glucosidase and α-amylase are presented in Table 1. α-Amylase initiates starch digestion by hydrolysing internal α-1,4-glycosidic bonds, producing smaller oligosaccharides and maltose. These products are subsequently hydrolysed by intestinal α-glucosidase into absorbable glucose. Therefore, inhibition of these enzymes may delay carbohydrate digestion and glucose release, thereby potentially reducing the postprandial rise in blood glucose [19].

In this study, the NADES extract exhibited significantly stronger inhibition of both enzymes than the water extract, as indicated by its lower IC_50_ values (*p* < 0.05). The IC_50_ values of the NADES and water extracts against α-glucosidase were 15.331 ± 0.393 and 17.536 ± 0.288 mg/mL, respectively. Similarly, the NADES extract showed a lower IC_50_ value against α-amylase (116.530 ± 2.110 mg/mL) than the water extract (182.080 ± 1.740 mg/mL). However, both extracts were less potent than the reference compounds, as higher concentrations were required to achieve 50% inhibition. Quercetin showed an α-glucosidase IC_50_ value of 1.467 ± 0.100 µg/mL, while acarbose exhibited an α-Amylase IC_50_ value of 816.920 ± 3.820 µg/mL.

The starch–iodine method used in the present study measures the amount of unhydrolysed starch remaining after enzymatic digestion. This differs from the commonly used 3,5-dinitrosalicylic acid (DNSA) method, which measures the reducing sugars released during starch hydrolysis. A previous study reported substantially different quercetin IC_50_ values when using the DNSA and starch–iodine methods, at 210.0 and 4343.0 µg/mL, respectively. This difference indicates that the analytical method can substantially influence the apparent inhibitory activity, possibly because phenolic compounds may interfere with starch–iodine complex formation [20]. Methodological differences may therefore partly account for the relatively high α-amylase IC_50_ values obtained in the present study. Nevertheless, direct comparisons between α-amylase and α-glucosidase IC_50_ values remain limited because the assays involve different enzymes, substrates, reaction conditions, and detection principles.

A previous study on thinned young kiwifruit extracted using a choline chloride–glycerol NADES (1:2) reported considerably lower α-glucosidase IC_50_ values of 23.82–62.98 µg/mL [8]. Despite the use of a similar NADES system, differences in cultivar, maturity, growing conditions, and extraction procedures may account for the variation in inhibitory activity. The influence of fruit maturity is supported by a previous study reporting α-glucosidase IC_50_ values of 48.02–70.10 mg/mL across eight maturation stages of A. chinensis ‘Donghong’ [21]. Meanwhile, studies on α-amylase inhibition by SunGold kiwifruit remain limited.

The stronger enzyme-inhibitory activities of the NADES extract may be associated with differences in the phenolic compounds recovered by the two solvent systems. The biological activity of phenolic extracts depends not only on the overall abundance of phenolic compounds but also on the identity, relative abundance, structural characteristics, and interactions of individual constituents. Flavonoids can inhibit carbohydrate-hydrolysing enzymes, with their inhibitory activity influenced by structural features such as hydroxyl groups at the C3′, C4′, C5, and C7 positions and the C2–C3 double bond [22]. SunGold kiwifruit has been reported to contain flavan-3-ols such as catechin, epicatechin, and procyanidin B1 [8]. Accordingly, the phenolic profiles of the NADES and water extracts were further characterised using LC-MS/MS, as discussed in Section 3.4.

### 3.2. In Vitro Glucose Diffusion

The in vitro glucose diffusion assay was used as a comparative cell-free physicochemical model to evaluate the ability of the extracts to retard the passive movement of glucose across a semipermeable dialysis membrane. Glucose movement across the membrane is driven by the concentration difference between the internal and external media [23]. The glucose dialysis retardation index (GDRI) represents the percentage reduction in glucose diffusion relative to the control. Therefore, a higher GDRI indicates greater glucose diffusion-retarding capacity. Extract components may delay glucose diffusion through physical interactions, glucose entrapment, or increased viscosity [24].

As shown in Table 2, dialysate glucose concentrations increased with incubation time because glucose progressively crossed the dialysis membrane. Nevertheless, both extracts significantly reduced glucose diffusion compared with the control. At 180 min, the NADES extract produced a significantly lower dialysate glucose concentration (0.492 ± 0.002 mM) than the water extract (0.822 ± 0.016 mM) and control (1.324 ± 0.043 mM), indicating stronger glucose diffusion-retarding capacity.

The GDRI of the NADES extract increased from 35.37% at 30 min to 62.84% at 180 min, indicating a sustained effect over time. Conversely, the water extract reached its highest GDRI at 60 min (41.39%) before decreasing to 37.89% at 180 min. This difference may be associated with variations in the phenolic profiles extracted by the two solvent systems. A similar solvent-dependent effect was reported for *Caralluma europaea*, where the ethyl acetate extract significantly reduced glucose diffusion, whereas the aqueous extract showed no significant effect compared with the control [24]. This suggests that solvent properties may influence the composition and glucose-retarding activity of the resulting extracts.

The stronger effect of the NADES extract may also be partly associated with its higher viscosity, which could restrict glucose mobility and slow its movement towards the dialysis membrane [23]. However, because NADES and water solvent blanks were not evaluated, the respective contributions of the solvent matrix and extracted compounds could not be distinguished. Further investigation is therefore required, as studies directly comparing NADES and water extracts using this assay remain limited.

This dialysis-based assay evaluates passive glucose diffusion and does not reproduce physiological intestinal glucose transport involving enterocytes, sodium-dependent SGLT1 transport, facilitated GLUT2 transport, tight junctions, or gastrointestinal metabolism. Therefore, the results should not be interpreted as direct evidence of reduced intestinal glucose absorption.

### 3.3. Antidiabetic Activities of Fortified Yoghurts

The α-glucosidase and α-amylase inhibitory activities of the raw extracts and fortified yoghurts are presented in Table 3. Fortified yoghurts generally exhibited greater inhibition of both enzymes than the unfortified control. The activity observed in the control yoghurt may be attributed to bioactive peptides released through proteolysis during fermentation or metabolites produced by lactic acid bacteria, consistent with previous findings for plain yoghurt [25]. These peptides may bind to key residues within or near the active and substrate-binding regions of carbohydrate-hydrolysing enzymes, such as α-glucosidase and α-amylase, through hydrogen bonding and electrostatic interactions, thereby restricting substrate accessibility or interfering with catalytic activity [26].

NADES-fortified yoghurts generally exhibited higher inhibitory activities than the corresponding water extract treatments, reflecting the stronger intrinsic activity of the NADES extract. The raw NADES extract showed significantly higher α-glucosidase activity (4.568 ± 0.130 mg QE/g) and α-amylase activity (370.500 ± 3.600 mg AE/g) than the water extract (3.359 ± 0.086 mg QE/g and 361.470 ± 2.930 mg AE/g, respectively). Increasing fortification concentration significantly enhanced the inhibition of both enzymes. However, signs of plateauing were observed only in selected α-glucosidase treatments, particularly between POS 20% and POS 30% NADES and between POS 30% water and the raw water extract, as indicated by the absence of significant differences between these treatments.

Fortification stage also significantly influenced both inhibitory activities, with POS treatments generally showing higher values than PRE treatments. Post-fermentation addition may better preserve enzyme-inhibitory compounds because they are less exposed to low pH, microbial enzymes, or protein–phenolic interactions during fermentation [27]. In addition, changes in phenolic structure and matrix interactions during fermentation may affect bioactivity, as hydroxylation, glycosylation, methylation, planarity, galloylation, and degree of polymerisation can influence polyphenol binding affinity toward α-amylase and α-glucosidase [28].

The interaction of the main effects was further evaluated through three-way ANOVA, as shown in Table 4. Extract type, fortification stage, and concentration had significant main effects on both α-glucosidase and α-amylase inhibitory activities (*p* < 0.05). This indicates that the antidiabetic activities of fortified yoghurts were independently influenced by the type of extract used, the timing of fortification, and the concentration of extract added. Significant interaction effects were also observed, showing that the effect of one factor depended on the level of another factor. For α-glucosidase activity, all two-way and three-way interactions were significant (*p* < 0.05), indicating that the response was strongly affected by the combined effects of extract type, stage, and concentration. Meanwhile, for α-amylase activity, most interactions were significant, except for stage x concentration (*p* = 0.266), suggesting that the concentration effect on α-amylase activity was relatively consistent between PRE and POS treatments.

### 3.4. Quantification of Phenolic Compounds from SunGold Kiwifruit Extracts

To compare the phenolic profiles and extraction selectivity of NADES and water, selected phenolic compounds were quantified by LC-MS/MS against their corresponding authentic reference standards. As shown in Table 5, the NADES extract was dominated by epicatechin, epigallocatechin, and catechin, with concentrations of 353.130 ± 1.920, 147.748 ± 1.334, and 36.000 ± 3.030 μg/g, respectively. In contrast, the water extract was mainly characterised by caffeic acid and chlorogenic acid, with concentrations of 85.330 ± 3.030 and 15.600 ± 0.150 μg/g, respectively. Minor compounds, including gallic acid and quercetin, were detected at lower levels. These compositional differences indicate that the choline chloride–glycerol NADES showed greater selectivity toward flavan-3-ols, particularly catechin, epicatechin, and epigallocatechin, than water under the tested extraction conditions.

Water extract was relatively more effective in recovering caffeic acid and chlorogenic acid, which may be attributed to the polar nature of these phenolic acids. Chlorogenic acids are esters formed between quinic acid and hydroxycinnamic acids, including caffeic acid, and their physicochemical properties are strongly influenced by the number and position of hydroxyl groups [29]. Hydroxyl groups increase molecular polarity, while the presence of hydroxyl and carboxyl groups contributes to the water-soluble nature of chlorogenic acid [30]. Therefore, the higher recovery of caffeic acid and chlorogenic acid in the water extract may be associated with their favourable solubility in aqueous media and hydrogen-bonding interactions with water.

In contrast, the choline chloride:glycerol NADES may favour the recovery of flavan-3-ols due to its hydrogen-bonding network and less purely aqueous environment. Previous study reported that choline chloride:glycerol was the most suitable NADES for extracting major catechins, including EGC, EC, EGCG, and ECG from green tea compared to ethanol and hot-water-based extraction [31]. This may be attributed to the supramolecular hydrogen-bonding structure of NADES, as excessive water addition can disrupt the NADES network and reduce its characteristic solvent properties that support the extraction of flavan-3-ols. Furthermore, choline chloride–glycerol NADES has been reported to form hydrogen bonds with catechins, supporting its compatibility with flavan-3-ol compounds [32]. Quercetin was detected only at low levels, likely due to its lower polarity and limited solubility in highly aqueous systems [33]. Its detection in the NADES extract suggests that NADES consisting of choline chloride:glycerol may improve the recovery of less water-soluble phenolics compared with water.

In NADES-fortified yoghurts, epicatechin, epigallocatechin, and catechin ranged from 1.497 to 15.910, 0.253 to 7.558, and 0.686 to 3.059 μg/g, respectively, while water-fortified yoghurts were mainly characterised by caffeic acid (0.813–4.182 μg/g) and chlorogenic acid (0.042–0.165 μg/g). These values were lower than in the corresponding raw extracts, likely due to dilution and possible interactions with the yoghurt matrix. Milk proteins, particularly casein, can bind polyphenols through hydrogen bonding and hydrophobic interactions, which may reduce the amount of free extractable phenolics [34].

Low levels of gallic acid, chlorogenic acid, caffeic acid, and catechin were also detected in the control yoghurt. The detection of low levels of phenolic-related compounds in control yoghurt by LC-MS may be attributed to endogenous milk-derived metabolites rather than the added kiwifruit extract. Previous LC-MS-based metabolomics studies have shown that milk can contain phenolic metabolites originating from animal feed, ruminal or intestinal microbial metabolism, and amino acid catabolism [35]. During yoghurt fermentation, bacterial proteolysis may further release small peptides and aromatic metabolites, which could contribute to the low-level phenolic-related signals observed in plain yoghurt [36].

Overall, extract type significantly influenced the phenolic profile of fortified yoghurts. NADES-fortified yoghurts were characterised by higher flavan-3-ols, particularly epicatechin, epigallocatechin, and catechin, whereas water-fortified yoghurts contained higher caffeic acid, chlorogenic acid, and gallic acid, reflecting the intrinsic profiles of the extracts. Phenolic levels generally increased with fortification concentration, although not all increases were statistically significant. Significant increases were mainly observed for PRE 30% water in gallic acid and caffeic acid, and for PRE 20% and PRE 30% NADES in catechin. In contrast, chlorogenic acid in all fortified yoghurts did not differ significantly from the control. This suggests that some compounds were present at low levels or were affected by matrix interactions, making them difficult to distinguish statistically from the control yoghurt.

In terms of fortification stage, PRE yoghurts generally showed higher phenolic concentrations than POS yoghurts. However, this difference was compound-dependent and was statistically significant mainly for epicatechin and epigallocatechin in NADES-fortified yoghurts, and for gallic acid in 30% water-fortified yoghurt. This may be related to the longer exposure of phenolic compounds to yoghurt fermentation when extracts were added before incubation. Starter cultures and increasing acidity can promote the hydrolysis of flavonoid glycosides and the cleavage of larger polyphenols into smaller phenolic acids, which may alter their stability, solubility, and extractability. Such transformations may either activate previously bound compounds or reduce specific phenolic forms, depending on the compound structure and yoghurt matrix interactions [12,37].

Although direct statistical correlation could not be established due to the limited number of comparable sample groups, the stronger antidiabetic activities of the NADES SunGold kiwifruit extract and NADES-fortified yoghurts may be partly explained by their phenolic profiles. NADES samples contained higher levels of flavan-3-ols, particularly catechin, epicatechin, and epigallocatechin, while epicatechin and epigallocatechin were not detected in the water extract or water-fortified yoghurts. In addition, the total quantified phenolic compounds were higher in the NADES extract (555.938 μg/g) than in the water extract (103.628 μg/g), although the water extract contained higher amounts of caffeic acid, chlorogenic acid, and gallic acid. This suggests that the relatively stronger in vitro enzyme-inhibitory activity of the NADES samples may be associated with differences in phenolic composition, particularly their greater abundance of flavan-3-ols.

Flavan-3-ols have been reported to contribute to antidiabetic effects through multiple mechanisms, including inhibition of carbohydrate-digesting enzymes, reduction in intestinal glucose absorption, improvement of glucose uptake, and modulation of insulin-signalling pathways [38]. Catechin and epicatechin, in particular, were reported to act as reversible and competitive α-glucosidase inhibitors, with IC_50_ values of 1.12 ± 0.03 and 0.95 ± 0.02 µM, respectively, which were substantially lower than that of acarbose (1250.11 ± 35.63 µM) [39]. Their inhibitory effects were associated with enzyme binding through electrostatic forces and hydrogen-bond interactions, particularly involving hydroxyl groups on the flavan-3-ol structure.

Epigallocatechin may also contribute to the observed antidiabetic activities. Previous studies reported that epigallocatechin inhibited both α-glucosidase and α-amylase in a non-competitive manner, with IC_50_ values of 1.04 ± 0.006 and 1.36 ± 0.006 mg/mL, respectively. Although these values were higher than those of acarbose against α-glucosidase (0.71 ± 0.27 mg/mL) and α-amylase (0.37 ± 0.31 mg/mL), epigallocatechin still showed measurable inhibitory activity. Its inhibition was attributed to hydrogen bonding and hydrophobic interactions with enzyme residues, which altered enzyme conformation and reduced enzyme activity [40].

Nevertheless, the antidiabetic activity of polyphenols should not be attributed to a single compound or pathway, but rather to the combined contribution of multiple compounds with specific structural features [41]. For carbohydrate-hydrolysing enzyme inhibition, structural features such as catechol groups on the B-ring can enhance enzyme binding, whereas methoxylation and glycosylation may reduce inhibitory activity [42]. Molecular planarity, electron delocalisation, hydroxyl availability, hydrogen bonding, and π–π interactions can also affect binding strength to enzyme active sites [43]. Therefore, further correlation studies involving a larger number of extract types or treatment groups should be conducted to confirm the relationship between individual phenolic compounds and antidiabetic activities. In addition, purified compound assays or compound combination studies would be useful to determine the specific contribution and possible synergistic effects of catechin, epicatechin, epigallocatechin, and other phenolics.

The yoghurt formulations evaluated in this study were experimental prototypes and were not demonstrated to be safe for direct commercial consumption. The concentration of choline chloride or choline in the final products was not analytically determined, and toxicological and sensory assessments were not performed. Further studies should quantify residual NADES components, establish appropriate fortification and serving levels, and evaluate safety before practical food application.

## 4. Conclusions

This study demonstrated that phenolic compounds extracted from SunGold kiwifruit using a choline chloride–glycerol-based NADES exhibited measurable in vitro antidiabetic activity. Compared with the water extract, the NADES extract showed stronger α-glucosidase and α-amylase inhibitory activities and greater glucose diffusion retardation under the tested conditions. The incorporation of the kiwifruit extracts into yoghurt further enhanced the enzyme-inhibitory activity relative to the unfortified control, with the effects influenced by extract type, concentration, and fortification stage. In particular, NADES-fortified yoghurts, especially at higher concentrations, generally exhibited greater enzyme-inhibitory activity than the corresponding water-fortified yoghurts.

LC-MS/MS analysis demonstrated extraction-dependent differences in phenolic composition. The NADES extract showed greater selectivity toward flavan-3-ols, particularly catechin, epicatechin, and epigallocatechin, whereas the water extract contained higher concentrations of caffeic acid, chlorogenic acid, and gallic acid. These compositional differences may partly contribute to the relatively stronger in vitro activity of the NADES extract, although the contributions of individual compounds require further investigation. Nevertheless, the relatively high α-amylase IC_50_ values indicate modest absolute inhibitory potency, and the dialysis assay represents only passive glucose diffusion rather than physiological intestinal absorption. Therefore, these findings should be regarded as preliminary comparative evidence rather than confirmation of glycemic management efficacy or immediate industrial applicability. Further studies should evaluate compound-specific activity, bioaccessibility, safety, sensory acceptability, storage stability, and in vivo efficacy before the practical application of these fortified yoghurts.

## Figures and Tables

**Table 1:** IC_50_ values of α-glucosidase and α-amylase inhibition by NADES and water extracts and reference inhibitors.

Sample	IC_50_ α-Glucosidase	IC_50_ α-Amylase
Quercetin	1.467 ± 0.110 ^c^ (µg/mL)	-
Acarbose	-	816.920 ± 3.820 ^a^ (µg/mL)
NADES extract	15.331 ± 0.393 ^b^ (mg/mL)	116.530 ± 2.110 ^c^ (mg/mL)
Water extract	17.536 ± 0.288 ^a^ (mg/mL)	182.080 ± 1.740 ^b^ (mg/mL)

Values are expressed as mean ± SD (n = 3). Within each column, means with different superscript letters are significantly different according to Tukey’s test (*p* < 0.05). Lower IC_50_ values indicate stronger inhibitory activity. “-“ indicates not tested.

**Table 2:** Glucose concentration in the dialysate and glucose dialysis retardation index (GDRI) of NADES and water extracts during in vitro glucose diffusion.

Sample	30 min (mM)	60 min (mM)	120 min (mM)	180 min (mM)
Control	0.435 ± 0.004 ^a^	0.765 ± 0.010 ^a^	1.052 ± 0.020 ^a^	1.324 ± 0.043 ^a^
NADES extract	0.281 ± 0.021 ^b^ (35.37%)	0.360 ± 0.020 ^b^ (52.91%)	0.447 ± 0.005 ^b^ (57.49%)	0.492 ± 0.002 ^b^ (62.84%)
Water extract	0.394 ± 0.007 ^c^ (9.47%)	0.448 ± 0.010 ^c^ (41.39%)	0.621 ± 0.003 ^c^ (41.02%)	0.822 ± 0.016 ^c^ (37.89%)

Values are expressed as mean ± SD (n = 3). Within each column, means with different superscript letters are significantly different according to Tukey’s test (*p* < 0.05). GDRI values (%) are presented in parentheses.

**Table 3:** α-Glucosidase and α-amylase inhibitory activities of SunGold kiwifruit extracts and fortified yoghurts.

Sample	α-Glucosidase Activity (mg QE/g)	α-Amylase (mg AE/g)
NADES extract	4.568 ± 0.130 ^a^	370.500 ± 3.600 ^a^
Water extract	3.359 ± 0.086 ^cd^	361.470 ± 2.930 ^b^
Control	1.352 ± 0.099 ^h^	18.201 ± 1.355 ^n^
PRE 10% NADES	2.420 ± 0.113 ^f^	32.923 ± 1.583 ^k^
PRE 20% NADES	3.312 ± 0.047 ^cd^	59.397 ± 2.157 ^g^
PRE 30% NADES	3.577 ± 0.050 ^b^	99.262 ± 0.817 ^d^
PRE 10% Water	1.633 ± 0.117 ^g^	22.552 ± 0.655 ^m^
PRE 20% Water	2.541 ± 0.096 ^f^	39.153 ± 1.768 ^j^
PRE 30% Water	3.198 ± 0.092 ^d^	66.758 ± 2.164 ^f^
POS 10% NADES	2.958 ± 0.036 ^e^	45.707 ± 1.913 ^i^
POS 20% NADES	3.472 ± 0.039 ^bc^	69.286 ± 1.689 ^f^
POS 30% NADES	3.615 ± 0.032 ^b^	111.186 ± 0.541 ^c^
POS 10% Water	1.706 ± 0.058 ^g^	28.252 ± 1.833 ^l^
POS 20% Water	2.811 ± 0.091 ^e^	49.816 ± 1.430 ^h^
POS 30% Water	3.335 ± 0.118 ^cd^	74.776 ± 1.096 ^e^

Values are expressed as mean ± SD (n = 6). Within each column, means that do not share a superscript letter are significantly different according to Tukey’s test (*p* < 0.05). The control refers to unfortified yoghurt prepared under the same fermentation conditions without the addition of NADES or water extract.

**Table 4:** Three-way ANOVA results for the antidiabetic activities of fortified yoghurt.

Response	Extract Type	Stage	Concentration	Extract × Stage	Extract × Concentration	Stage × Concentration	Extract × Stage × Concentration
α-Glucosidase activity	<0.001	<0.001	<0.001	0.039	<0.001	<0.001	<0.001
α-Amylase activity	<0.001	<0.001	<0.001	0.012	<0.001	0.266	0.038

Values represent *p*-values obtained from three-way ANOVA. Significant effects were considered at *p* < 0.05. Extract type refers to NADES and water extracts; stage refers to PRE and POS fortification; concentration refers to 10%, 20%, and 30% extract addition.

**Table 5:** Phenolic compounds in SunGold kiwifruit extracts and fortified yoghurts determined using LC-MS/MS.

Sample	Gallic Acid (μg/g)	Chlorogenic Acid (μg/g)	Catechin (μg/g)	Caffeic Acid (μg/g)	Quercetin (μg/g)	Epicatechin (μg/g)	Epigallocatechin (μg/g)
NADES extract	0.155 ± 0.038 ^bc^	10.646 ± 0.843 ^b^	36.000 ± 3.030 ^a^	7.440 ± 2.180 ^b^	0.819 ± 0.085 ^a^	353.130 ± 1.920 ^a^	147.748 ± 1.334 ^a^
Water extract	0.600 ± 0.150 ^a^	15.600 ± 0.150 ^a^	2.098 ± 0.687 ^bc^	85.330 ± 3.030 ^a^	nd	nd	nd
Control	0.012 ± 0.000 ^c^	0.004 ± 0.002 ^c^	0.013 ± 0.012 ^c^	0.008 ± 0.005 ^d^	nd	nd	nd
PRE 10% NADES	0.025 ± 0.005 ^c^	0.138 ± 0.004 ^c^	1.765 ± 0.068 ^bc^	0.093 ± 0.029 ^d^	0.034 ± 0.030 ^b^	7.348 ± 0.314 ^c^	1.263 ± 0.091 ^d^
PRE 20% NADES	0.036 ± 0.012 ^c^	0.362 ± 0.025 ^c^	2.890 ± 0.289 ^b^	0.194 ± 0.041 ^d^	0.038 ± 0.023 ^b^	13.564 ± 1.032 ^b^	3.984 ± 0.413 ^c^
PRE 30% NADES	0.061 ± 0.074 ^c^	0.482 ± 0.110 ^c^	3.059 ± 0.228 ^b^	0.232 ± 0.055 ^d^	0.087 ± 0.014 ^b^	15.910 ± 2.310 ^b^	7.558 ± 0.628 ^b^
PRE 10% Water	0.170 ± 0.038 ^bc^	0.078 ± 0.013 ^c^	0.448 ± 0.036 ^c^	0.892 ± 0.021 ^d^	nd	nd	nd
PRE 20% Water	0.245 ± 0.007 ^bc^	0.119 ± 0.018 ^c^	0.900 ± 0.125 ^bc^	2.456 ± 0.232 ^cd^	nd	nd	nd
PRE 30% Water	0.448 ± 0.312 ^ab^	0.165 ± 0.009 ^c^	1.506 ± 0.030 ^bc^	4.182 ± 0.415 ^c^	nd	nd	nd
POS 10% NADES	0.099 ± 0.120 ^c^	0.036 ± 0.002 ^c^	0.686 ± 0.086 ^bc^	0.070 ± 0.005 ^d^	0.008 ± 0.002 ^b^	1.497 ± 0.210 ^e^	0.253 ± 0.023 ^d^
POS 20% NADES	0.014 ± 0.002 ^c^	0.163 ± 0.012 ^c^	1.529 ± 0.190 ^bc^	0.175 ± 0.023 ^d^	0.020 ± 0.006 ^b^	3.475 ± 0.465 ^de^	0.319 ± 0.075 ^d^
POS 30% NADES	0.039 ± 0.005 ^c^	0.307 ± 0.038 ^c^	2.151 ± 0.075 ^bc^	0.212 ± 0.036 ^d^	0.047 ± 0.003 ^b^	6.274 ± 0.783 ^cd^	1.076 ± 0.159 ^d^
POS 10% Water	0.047 ± 0.008 ^c^	0.042 ± 0.000 ^c^	0.330 ± 0.050 ^c^	0.813 ± 0.003 ^d^	nd	nd	nd
POS 20% Water	0.077 ± 0.005 ^c^	0.089 ± 0.006 ^c^	0.660 ± 0.047 ^bc^	1.528 ± 0.126 ^cd^	nd	nd	nd
POS 30% Water	0.099 ± 0.119 ^c^	0.161 ± 0.009 ^c^	0.949 ± 0.158 ^bc^	2.557 ± 0.104 ^cd^	nd	nd	nd

Values are expressed as mean ± SD (n = 3). Within each column, means that do not share a superscript letter are significantly different according to Tukey’s test (*p* < 0.05). “nd” indicates not detected.

## Data Availability

The original contributions presented in this study are included in the article. Further inquiries can be directed to the corresponding author.

## Abbreviations

The following abbreviations are used in this manuscript:

AE	Acarbose equivalent
ANOVA	Analysis of variance
CAE	Caffeic acid equivalent
CE	Catechin equivalent
ChAE	Chlorogenic acid equivalent
DMSO	Dimethyl sulfoxide
DNSA	3,5-Dinitrosalicylic acid
ECE	Epicatechin equivalent
EGCE	Epigallocatechin equivalent
GAE	Gallic acid equivalent
GDRI	Glucose dialysis retardation index
H-ESI	Heated electrospray ionisation
HCl	Hydrochloric acid
HPLC	High-performance liquid chromatography
IC_50_	Half-maximal inhibitory concentration
LC-MS/MS	Liquid chromatography–tandem mass spectrometry
NADES	Natural deep eutectic solvent
nd	Not detected
*p*-NPG	p-Nitrophenyl-α-D-glucopyranoside
POS	Post-fermentation fortification
PRE	Pre-fermentation fortification
QE	Quercetin equivalent
SD	Standard deviation
SPE	Solid-phase extraction
T2DM	Type 2 diabetes mellitus
UHPLC	Ultra-high-performance liquid chromatography
UV-Vis	Ultraviolet–visible
